# Congenital Bronchial Atresia Mimicking Pulmonary Sequestration in the Lower Lobe: A Case Report

**DOI:** 10.1002/rcr2.70712

**Published:** 2026-09-07

**Authors:** Shohei Mitsumata, Kensuke Midorikawa, Kentaro Wakamatsu, Shiro Kaneda, Takuro Futamata, Tsuyoshi Iwanaka, Yuichiro Ueda, So Miyahara, Yoshiko Masuda, Hiroyasu Nakashima, Toshihiko Sato

**Affiliations:** ^1^ Department of General Thoracic, Breast and Pediatric Surgery Fukuoka University School of Medicine and Hospital Fukuoka Japan; ^2^ National Hospital Organization Omuta National Hospital Omuta Japan

**Keywords:** bronchial atresia, bronchocele, congenital lung anomaly, lung infection, pulmonary sequestration

## Abstract

Congenital bronchial atresia (CBA) is a rare airway anomaly that may radiologically resemble other congenital pulmonary lesions, particularly when it occurs in the lower lobes. A 41‐year‐old woman was referred for surgical treatment of suspected pulmonary sequestration based on imaging findings. Computed tomography (CT) at the referring hospital showed a cavitary lesion with fluid retention in Segment 7 (S7) of the right lower lobe, with surrounding ground‐glass opacity, suggesting infection associated with pulmonary sequestration. Re‐evaluation at our institution identified the lesion as a bronchocele with focal hyperinflation, interruption of the segmental bronchus (B7), and a normally distributed pulmonary artery (A7) without aberrant systemic supply. Three‐dimensional CT clearly demonstrated these bronchovascular features and supported the diagnosis of CBA. Thoracoscopic right lower lobectomy was performed, and histopathological findings confirmed the diagnosis of CBA. This case highlights the importance of systematic evaluation of bronchial continuity and vascular supply in congenital lower‐lobe lesions, for which three‐dimensional CT reconstruction may be useful.

## Graphical abstract

Lower‐lobe congenital bronchial atresia may resemble pulmonary sequestration, particularly in the presence of recurrent infection and cavitary changes. Systematic evaluation of bronchial continuity and vascular supply using three‐dimensional CT was useful for accurate diagnosis and surgical decision‐making.

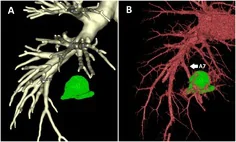

## 1 Introduction

Congenital bronchial atresia (CBA) is a rare developmental anomaly characterized by focal interruption of a lobar, segmental, or subsegmental bronchus with preservation of the distal bronchial tree. Obstruction of the distal airway leads to mucus retention, resulting in a bronchocele or mucocele, while collateral ventilation causes hyperinflation of the affected lung parenchyma. Therefore, the combination of a bronchocele and distal hyperinflation is considered the characteristic radiologic hallmark of CBA [1, 2, 3, 4].

CBA is often detected incidentally, although some patients present with recurrent infection or other respiratory symptoms [1, 2, 3, 4]. Computed tomography (CT) usually enables diagnosis by demonstrating bronchocele formation, abrupt bronchial interruption and adjacent hyperlucent or emphysematous changes [1, 2, 3, 4]. However, diagnosis may be challenging when CBA occurs in the lower lobes or is accompanied by infection‐related inflammatory changes.

Pulmonary sequestration is an important differential diagnosis of lower‐lobe congenital pulmonary lesions and is characterized by the absence of normal bronchial communication and the presence of aberrant systemic arterial supply, typically arising from the aorta [5, 6]. Because lower‐lobe CBA may also present with recurrent infection and cystic or cavitary changes, differentiation from pulmonary sequestration can occasionally be difficult.

Herein, we report a rare case of CBA confined to Segment 7 of the right lower lobe that radiologically resembled pulmonary sequestration. This case emphasizes the importance of systematic evaluation of bronchial continuity and vascular supply in congenital lower‐lobe lesions.

## 2 Case Report

A 41‐year‐old woman was referred to our department for surgical treatment of suspected pulmonary sequestration. She presented with persistent low‐grade fever and purulent sputum. Chest radiography revealed an abnormal shadow in the right lung field, and CT demonstrated a cavitary lesion with internal fluid retention in the right lower lobe, accompanied by surrounding ground‐glass opacity suggestive of inflammation. She had experienced several similar episodes annually that improved with oral antibiotics. She had no significant medical or smoking history.

**FIGURE 1:**
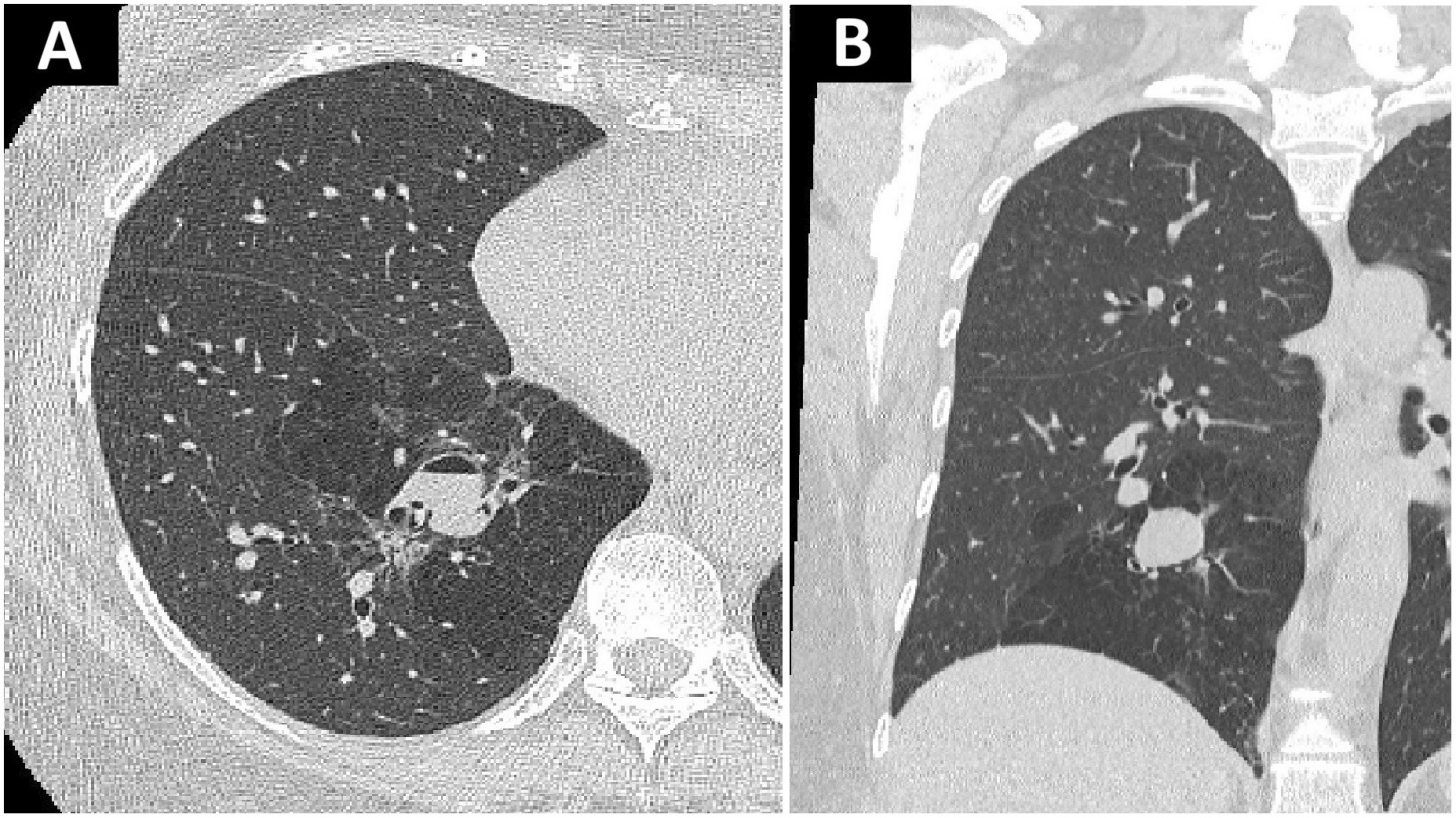
Computed tomography findings at presentation. (A) Axial CT image showing a cavitary lesion with internal fluid retention in Segment 7 (S7) of the right lower lobe, consistent with a bronchocele, accompanied by surrounding ground‐glass opacity suggestive of inflammation. (B) Coronal CT image demonstrating localized hyperinflation confined to S7.

**FIGURE 2:**
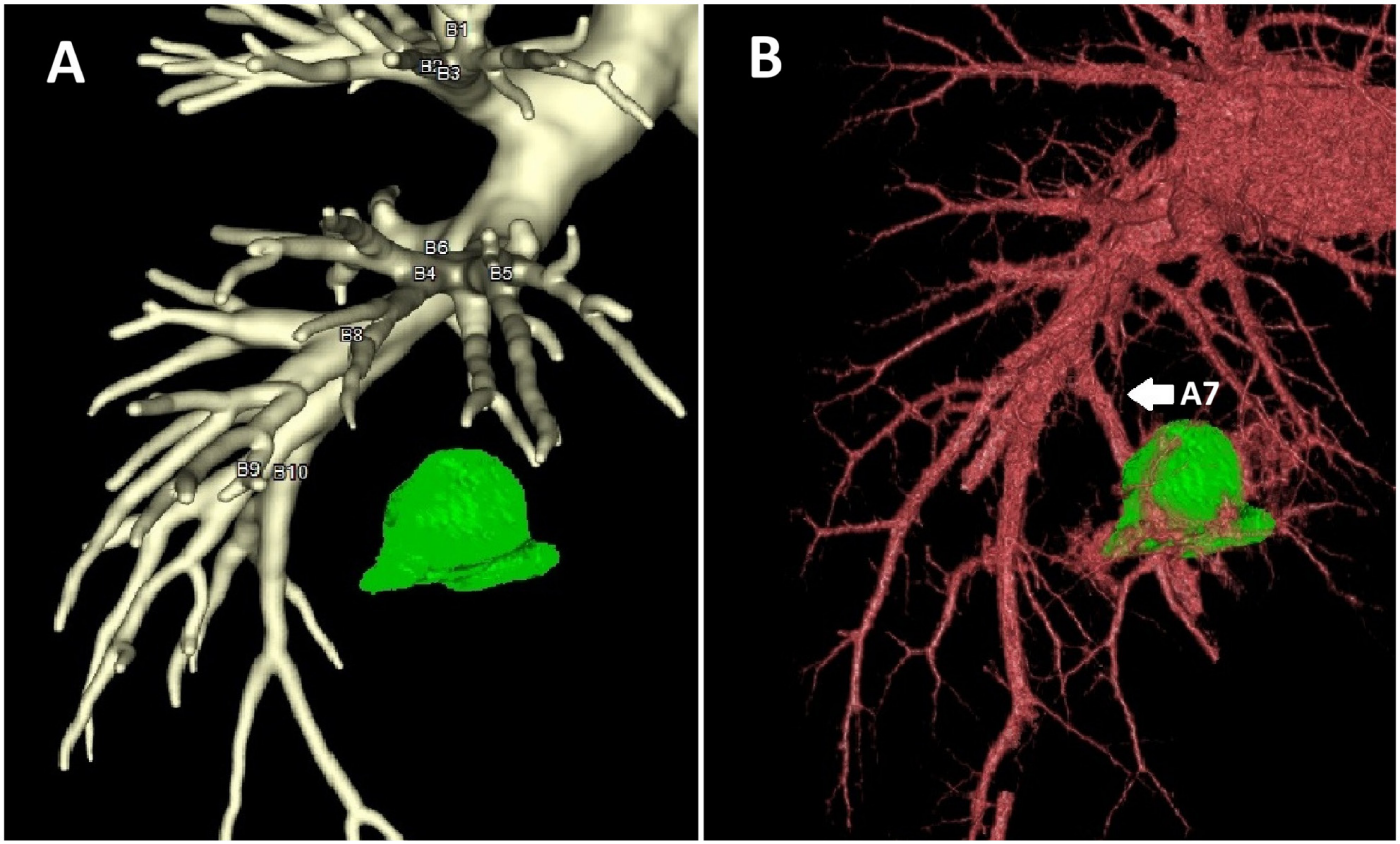
Three‐dimensional computed tomography reconstruction of the bronchial and pulmonary arterial anatomy. (A) Bronchial reconstruction demonstrating interruption of the right B7 bronchus. (B) Pulmonary arterial reconstruction showing a normally branching segmental artery (A7) (arrow) supplying the lesion.

After antibiotic therapy, the inflammatory findings improved, and she was referred for surgical management. Repeat CT at our institution demonstrated segmental emphysematous change localized mainly to Segment 7 (S7) of the right lower lobe, a cavitary lesion with internal fluid retention, and faint residual ground‐glass opacity (Figure 1). Interruption of the right B7 bronchus was identified. No aberrant systemic arterial supply from the aorta was observed, and the pulmonary artery showed a normal branching pattern with a segmental artery (A7) supplying the lesion. These findings suggested CBA rather than pulmonary sequestration, and the cavitary lesion was considered a bronchocele. Three‐dimensional CT reconstruction clearly demonstrated interruption of B7 and the normal distribution of A7 (Figure 2). The pulmonary venous anatomy was normal.

Based on the clinical course, the lesion was considered responsible for recurrent infection, and surgical resection was planned. Pulmonary function was within normal limits, and laboratory findings showed only mild inflammatory change.

**FIGURE 3:**
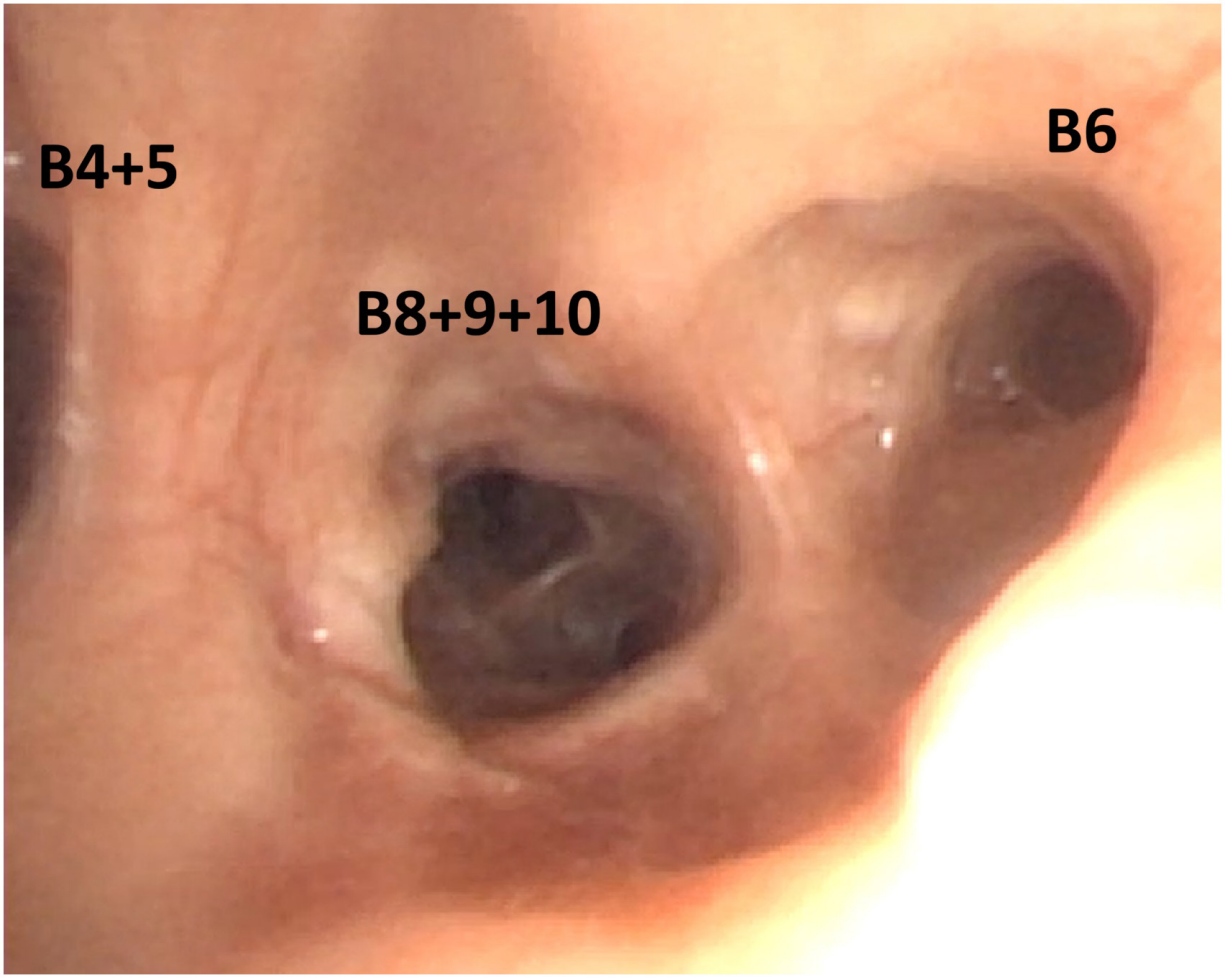
Intraoperative bronchoscopic findings. Bronchoscopic view at the level of the right intermediate bronchus demonstrating no identifiable orifice of the B7 bronchus. The orifices of B6 and the basal bronchi (B8–10) are identified.

Although the lesion was centered in S7, hyperinflation extended close to the basal bronchial bifurcation, and inflammatory changes had involved the adjacent segments on previous CT scans. Therefore, complete resection by segmentectomy was considered difficult, and thoracoscopic right lower lobectomy was performed. Intraoperative bronchoscopy demonstrated interruption of the B7 bronchus at the intermediate bronchial level (Figure 3). The operation time was 123 min with minimal blood loss. The postoperative course was uneventful, and the patient was discharged on postoperative Day 8.

**FIGURE 4:**
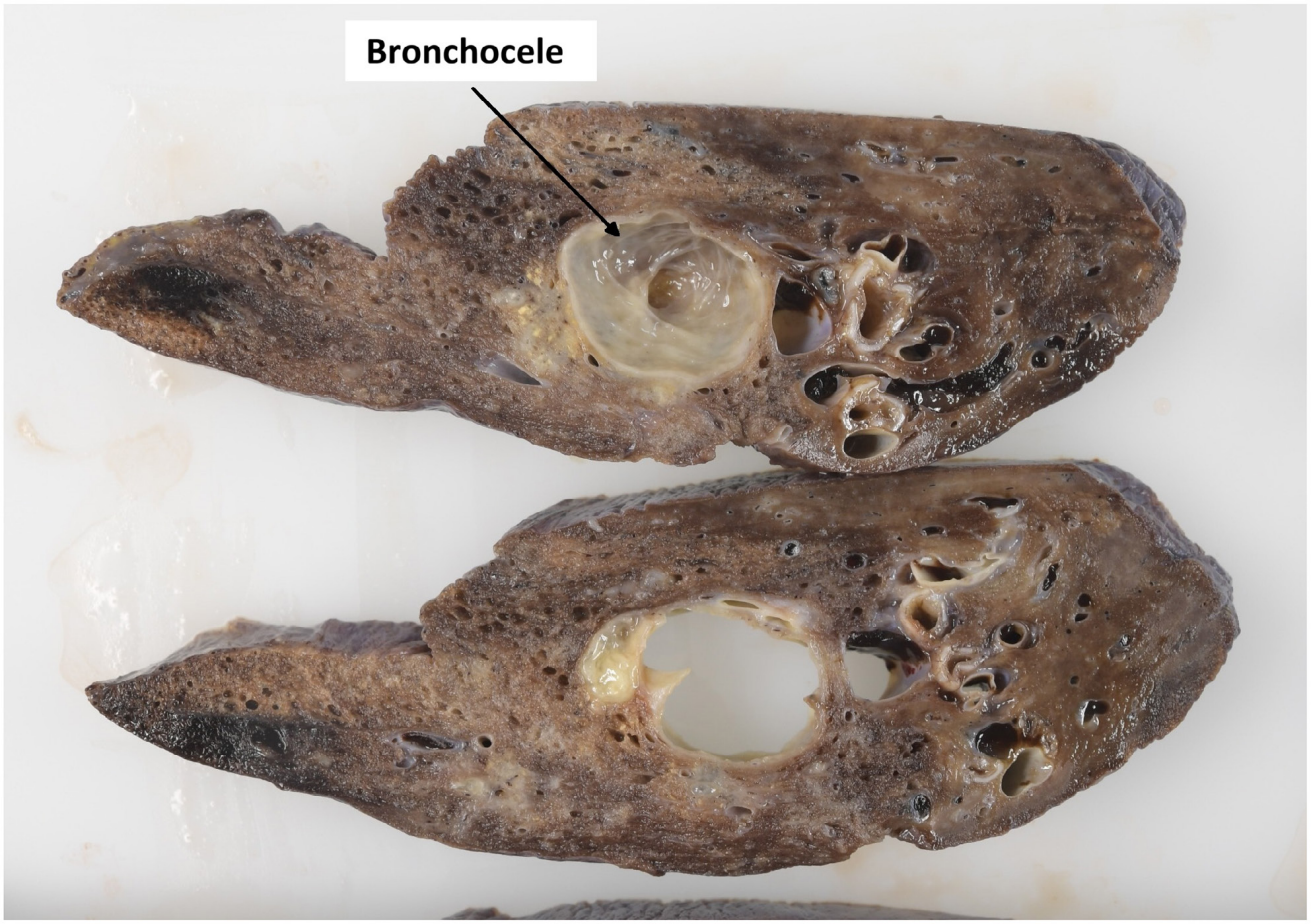
Macroscopic pathological findings. A cavitary lesion composed of mucus‐filled bronchial structures without communication with the central airway is observed (arrow), consistent with bronchocele associated with congenital bronchial atresia.

Histopathological examination revealed a cavitary lesion composed of bronchial structures with mucus retention and no communication with the central airway, consistent with bronchocele associated with CBA (Figure 4). Based on the radiological, bronchoscopic and histopathological findings, the final diagnosis was CBA.

## 3 Discussion

The present case illustrates the importance of systematic anatomical evaluation in congenital lower‐lobe lesions. Although bronchocele and distal hyperinflation are characteristic radiological findings of CBA, lower‐lobe location, recurrent infection and cavitary appearance may cause CBA to radiologically resemble pulmonary sequestration. Therefore, evaluation of both bronchial continuity and vascular supply is essential for accurate differentiation.

CBA is a relatively rare congenital anomaly compared with pulmonary sequestration [1, 7]. While pulmonary sequestration accounts for approximately 0.15%–6.4% of congenital pulmonary malformations, CBA is less frequently encountered in clinical practice and is often detected incidentally [3, 7]. Furthermore, CBA most commonly involves the upper lobes, particularly the left upper lobe, whereas lower‐lobe involvement is uncommon in adult radiologic series [2, 8]. Although lower‐lobe cases have been reported, segmental bronchial atresia confined to a single basal segment, such as S7, is extremely rare [3]. In such cases, recurrent infection and cavitary or cystic changes may closely resemble intralobar pulmonary sequestration, making accurate diagnosis challenging.

The key distinction between CBA and pulmonary sequestration lies in bronchial and vascular anatomy. In CBA, the bronchus is interrupted proximally while the distal airway is preserved and filled with mucus, and the pulmonary arterial supply remains normal. In contrast, pulmonary sequestration is characterized by the absence of normal bronchial communication and the presence of aberrant systemic arterial supply, typically arising from the aorta [5, 6]. In the present case, three‐dimensional CT reconstruction clearly demonstrated interruption of the B7 bronchus and the presence of a normally distributed segmental artery (A7) without aberrant systemic arterial supply. These findings were essential for establishing the correct diagnosis of CBA.

Surgical resection is generally indicated in patients with CBA who develop recurrent infection or other symptoms [3, 9]. The choice of surgical procedure depends on the extent of the lesion and the condition of the surrounding lung parenchyma. Segmentectomy may be feasible in localized lesions, whereas lobectomy is often selected in cases with marked hyperinflation or associated infection [9, 10]. In the present case, although the lesion was centered in S7, hyperinflation extended close to the basal bronchial bifurcation and inflammatory changes had involved adjacent segments. Therefore, complete resection by segmentectomy was considered difficult, and thoracoscopic right lower lobectomy was selected.

Three‐dimensional CT may also be useful for surgical planning because it enables detailed evaluation of bronchovascular anatomy. Previous reports have suggested that such imaging is particularly valuable when sublobar resection is considered, as precise anatomical understanding is required for safe and appropriate resection [10].

In summary, lower‐lobe CBA may radiologically resemble pulmonary sequestration, particularly when accompanied by recurrent infection and cystic or cavitary changes. Systematic evaluation of bronchial continuity and vascular supply is important for accurate differentiation, and three‐dimensional CT reconstruction may be useful for visualizing these anatomical features and guiding surgical decision‐making.

## Author Contributions

Shohei Mitsumata contributed to study conception, data collection, image analysis and manuscript drafting. Kensuke Midorikawa contributed to study supervision and critical revision of the manuscript. Kentaro Wakamatsu contributed to radiological and pulmonological evaluation and manuscript revision. Shiro Kaneda, Takuro Futamata, Tsuyoshi Iwanaka, Yuichiro Ueda, So Miyahara, Yoshiko Masuda, Hiroyasu Nakashima and Toshihiko Sato contributed to clinical management, data interpretation and manuscript revision. All authors read and approved the final manuscript.

## Funding

The authors have nothing to report.

## Consent

The authors declare that written informed consent was obtained for the publication of this manuscript and accompanying images using the consent form provided by the Journal.

## Conflicts of Interest

The authors declare no conflicts of interest.

## Data Availability

The data that support the findings of this study are available from the corresponding author upon reasonable request.
